# Estimating mortality among inpatients with acute exacerbation of chronic obstructive pulmonary disease using registry data

**DOI:** 10.1038/s41533-020-0186-y

**Published:** 2020-06-16

**Authors:** Zhengcun Pei, Yixin Sun, Shengfeng Wang, Yahong Chen, Ting Yang, Kewu Huang, Yan Zhang, Yin Huang, Chen Wang, Siyan Zhan

**Affiliations:** 1 Department of Epidemiology and Biostatistics, School of Public Health, Peking University, Beijing, China; 2 Department of Pulmonary and Critical Care Medicine, Peking University Third Hospital, Beijing, China; 3 Department of Pulmonary and Critical Care Medicine, Center of Respiratory Medicine, China-Japan Friendship Hospital, Beijing, China; 4 National Clinical Research Center for Respiratory Diseases, Beijing, China; 5 Department of Pulmonary and Critical Care Medicine, Beijing ChaoYang Hospital, Beijing, China; 6 Department of Respiratory Medicine, Capital Medical University, Beijing, China; 7 Best-Road Medi-Tech (Beijing) Ltd, Beijing, China; 8 Beijing Natureself Technology Co. Ltd, Beijing, China; 9 Chinese Academy of Medical Sciences and Peking Union Medical College, Beijing, China

**Keywords:** Epidemiology, Chronic obstructive pulmonary disease

## Abstract

The study aimed to investigate the demographic characteristics, clinical features, diagnoses, and treatments of hospitalized exacerbation COPD patients, as well as their disease prognoses and economic costs. The study planned to enroll 7600 hospitalized patients (aged ≥18 years with main diagnosis as AECOPD). Study patients were recruited since September 2017, followed up with a 3-year observing period. In the baseline visit, information on demographic characteristics, clinical features, diagnoses, and treatments were collected. In the following visits, treatments and examinations, recurrence of AECOPD, re-admission to hospital, complications, and mortality were recorded. Several validated questionnaires were applied at specific visits. This study included data from 1 September 2017 until 31 December 2022. The data would be used to estimate all-cause mortality during hospital stay, AECOPD recurrence within 1 month after discharge, all-cause and cause-specific mortality, frequency of AECOPD recurrence, lung function, life quality, healthcare costs in the study period, etc.

## Introduction

Chronic obstructive pulmonary disease (COPD), characterized by persistent airflow limitation, is a common preventable and treatable disease and is the leading cause of mortality and morbidity worldwide^1^. More than three million deaths were caused by COPD each year^2,3^. The prevalence of COPD in adults was estimated to be 4–10% worldwide^4,5^, and 8.6% in China^6^, accounting for approximately 100 million COPD patients.

Acute exacerbation of COPD (AECOPD), defined by the Global Initiative for Chronic Obstructive Lung Disease, is an acute event characterized by a worsening of the patient’s respiratory symptoms that is beyond normal day-to-day variations and leads to a change in medication^7–9^; the disease burden and its exacerbations have been studied globally^3,10,11^; exacerbation is a signal of disease progression and a major cause of patient hospitalization.

The severity and frequency of exacerbations strongly correlated with patient prognosis, especially mortality, whereas prevention, early detection, and prompt treatment would predict a better prognosis^2^. However, as the mortality data with regard to AECOPD are limited for China, we speculated that there remains a gap between guidelines and clinical practices in China. This multicenter prospective patient registry study is launched to investigate the clinical features, treatments, and prognoses of AECOPD and meanwhile to build up a COPD management network.

The primary aim of this study was to assess the all-cause and cause-specific mortality among patients admitted to hospital for AECOPD and further to calculate re-admission rate caused again by AECOPD within 1 month after discharge. The secondary aims included analyses with regard to the 3-year follow-up after discharge, specifically all-cause and cause-specific mortality (at 1, 2, 3 years), recurrence of AECOPD (frequency of AECOPD recurrence, the date of first recurrence after discharge), lung function, and quality of life assessment, as well as healthcare costs.

## Results

The primary aim of this study was to assess the in-hospital mortality of AECOPD patients, to describe all-cause and cause-specific mortality, and further to calculate re-admission rate caused again by AECOPD within 1 month after discharge from hospital. The secondary aims included analyses with regard to the 3-year follow-up after discharge from hospital, specifically all-cause and cause-specific mortality (at 1, 2, 3 years), recurrence of AECOPD (frequency of AECOPD recurrence, the date of first recurrence after discharge), lung function, and quality of life assessment, as well as healthcare costs.

The data obtained were planned to be used to describe the all-cause and cause-specific mortality of AECOPD patients, re-admission rates at the time points of 1 month to 3 years after discharge from hospital, as well as the recurrence of AECOPD, lung function decline, and quality of life.

Mortality rate, recurrence rate, and re-admission rate were planned to be reported pooled and stratified by sex, age, area, disease severity, etc. Their potential risk factors and confounders in consideration included COPD severity (lung function, AECOPD symptoms), medical history (COPD history, frequency of hospitalization, tobacco exposure, etc.), comorbidities (respiratory diseases, cardiovascular diseases, metabolic diseases, digestive diseases, etc.), treatment (bronchodilators, glucocorticoids, antibiotics, oxygen therapy, ventilatory support, etc.), questionnaire, and scales (COPD assessment test (CAT), modified British Medical Research Council (mMRC), St. George’s respiratory questionnaire (SGRQ), hospital anxiety and depression scale (HADS)). The association between death event and its potential influential factors, the association between recurrence and readmission with their risk factors, the association between treatment and future outcomes, and similar associations of interest were planned to be assessed using multivariate linear/logistic regressions and Cox regression to assess time to death and its relevant factors. A mixed-effects model for longitudinal data analysis would be an optional choice to validate long-term effect of potential risk factors. In addition, comparative effectiveness research on different inpatient therapies (bronchodilators, glucocorticoids, antibiotics, oxygen therapy, ventilatory support) and prognoses (recovery, death, length of hospitalization), stable COPD management (pharmacological treatment, tobacco cessation, pulmonary rehabilitation, oxygen therapy), and disease progression (death, readmission, new comorbidities, lung function decline), as well as the impact of medication adherence on disease progression were planned to be compared after propensity score matching. Statistical significance were defined as the two-sided *p* value < 0.05. All the data were planned to be analyzed using the R software version 3.6, SAS V9.4 statistical package or STATA V15.0.

## Discussion

The ACURE (estimating mortality among inpatients with Acute exacerbation of Chronic obstrUctive pulmonary disease using REgistry data) study aimed to describe the overall clinical features and treatment procedures of AECOPD patients. The data obtained could be used to better understand the long-term outcome and risk factors of AECOPD and lung function declines.

As a pioneering study for proper measuring mortality of AECOPD patients all over the country, this study enabled our clinicians and researchers to address fundamental issues regarding the “real world” situation of AECOPD in China. It will also serve as a harmonized, evidence-based registry and platform for conducting future research, which will ultimately improve the management care provided to AECOPD patients.

The main limitations may be related to incompleteness of lung function data, as most exacerbation patients were not able to conduct a lung function test during hospital stay. We were aware of this limitation and asked the patient to provide lung function information before exacerbation or to measure lung function during stable phase of COPD as a substitution. Additional limitation is that selection bias may exist, as participating research centers are leading hospitals where proportions of severe patients are usually higher than normal population. Nevertheless, participant from diverse areas of China would represent a real nature of COPD progression in Chinese patients. Disease progression of mild, moderate, and severe COPD were all investigated and discussed, regardless of their actual proportion.

## Methods

**Fig. 1:**
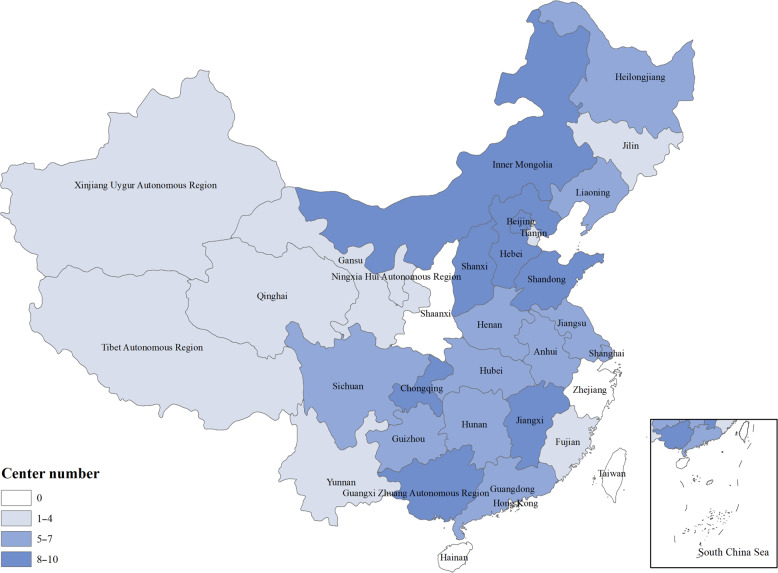
Distribution of participating centers of the ACURE study. White and blue colors denote different center numbers across provinces.

The ACURE study is an ongoing nationwide multicenter, observational patient registry in patients admitted to hospital for AECOPD, followed up with a 3-year observing period in a real-world setting. The first patient recruited in the database was recorded on 1 September 2017, the expected end of patient enrollment in all centers was December 2019, and the expected end of patient follow-up in all centers is December 2022. The study design schematic is shown in Table 1. Currently, 158 centers (hospitals) dispersing over 29 provinces of China participated in this study. The distribution of participating centers is shown in Fig. 1; each research center was coordinated by one national expert, who was responsible for local data collection and organization affairs that were related with this work. In addition, a steering committee was employed for the design and scientific integrity of the study. Table 2 shows the composition of our steering committee.

**Table 1:** Schematic diagram of the ACURE study.

Visit	Study period							
	Baseline survey	Follow-up survey						
	V0	V1	V2	V3	V4	V5	V6	V7
Time point	Admission–discharge	D30	M3	M6	M9	M12	M24	M36
Contact type	V	V	P	V	P	V	V	V
Eligibility screen	X							
Informed consent	X							
Demographics	X							
Medical history	X							
Personal history	X							
Treatment	X	X	X	X	X	X	X	X
Change of treatment for respiratory system		X	X	X	X	X	X	X
Physical examination	X							
Lung function test	X	X		X		X	X	X
Laboratory test	X	X		X		X	X	X
ECG	X							
Lung CT	X					X	X	X
UCG	X							
Pulmonary perfusion imaging	X							
Venous ultrasound of lower extremity	X							
PEACE questionnaire	X^a^	X	X	X	X	X	X	X
CAT questionnaire	X	X	X	X	X	X	X	X
mMRC questionnaire		X	X	X	X	X	X	X
SGRQ		X				X	X	X
HADS				X				
Prognosis (including death)	X	X		X		X	X	X
RICU/ICU admission	X							
Re-admission		X	X	X	X	X	X	X
AECOPD assessment		X	X	X	X	X	X	X
Lost to follow-up		X		X		X	X	X
Direct cost	X	X	X	X	X	X	X	X
Inhalation equipment				X				

*ACURE* estimating mortality among inpatients with Acute exacerbation of Chronic obstrUctive pulmonary disease using REgistry data, *AECOPD* acute exacerbation of chronic obstructive pulmonary disease, *CAT* COPD assessment test, *CT* computed tomography, *D30* 30 days after discharge, *ECG* electrocardiogram, *HADS* hospital anxiety and depression scale, *M3*, *M6*, *M9*, *M12* 3/6/9/12 months after discharge, *mMRC* modified British Medical Research Council, *P* phone call, *PEACE* questionnaire from the study by Zheng et al.^13^, *RICU* respiratory intensive care unit, *SGRQ* St. George’s respiratory questionnaire, *UCG* ultrasound cardiogram, *V* visit, *X* information collected.

^a^Everyday during hospitalization.

**Table 2:** Steering committee of the ACURE study.

Center	Province/city	Principal investigator/coordinator
Peking University Third University	Beijing	Yahong Chen, MD
Chinese PLA General Hospital	Beijing	Junchang Cui, MD
Peking Union Medical College Hospital	Beijing	Jinglan Wang, MD
Beijing Hospital	Beijing	Chun Pu, MSc
The Second Hospital of Hebei Medical University	Hebei	Xixin Yan, MD
The First Affiliated Hospital of Chongqing Medical University	Chongqing	Shuliang Guo, MD
West China Hospital Sichuan University	Sichuan	He Yu, MSc
Changhai Hospital	Shanghai	Yuchao Dong, MD
The Second Affiliated Hospital of Chongqing Medical University	Chongqing	Daoxin Wang, MD
The First Hospital of China Medical University	Liaoning	Hongwen Zhao, MD
General Hospital of East China	Jiangsu	Xiaoyong Xu, MD
China-Japan Friendship Hospital	Beijing	Ting Yang, MD

*ACURE* estimating mortality among inpatients with Acute exacerbation of Chronic obstrUctive pulmonary disease using REgistry data, *PLA* People’s Liberation Army.

All patients with AECOPD examined by a clinical physician were eligible to be enrolled in this study if they fulfill the following inclusion criteria: age ≥18 years and hospitalized patients with confirmed diagnosis of AECOPD. During recruitment, we confirmed AECOPD diagnosis based on personal history and clinical symptoms, as well as lung function level in recent 6 months, which was recorded in hospital information system or self-reported. For patients who had AECOPD for the first time or who had no lung function data, we confirmed their AECOPD diagnoses at follow-up lung function examinations, as it is not recommended to test lung function during acute exacerbations. Meanwhile, patients who were diagnosed as having active pulmonary tuberculosis or acute left heart failure and patients who were participating in clinical trials or intervention studies of drugs were excluded.

Patients were not directly involved in the design, development of research questions or outcomes of this study. Researchers or research assistants interpreted the questions to the participants and recorded the answers. The results of the ACURE study will be available for the public. All participating patients were requested to provide written informed consent.

The study had complied with all relevant ethical regulations. The study protocol had been approved by the ethics committee of China-Japan Friendship Hospital (approval number: 2015-88). Informed consent was obtained from all patients of the study. The rights, safety, and well-being of clinical investigation subjects were protected according to the ethical principles of the Declaration of Helsinki. This study was registered in Clinicaltrials.gov with the identifier NCT02657525.

The study protocol was designed to collect all clinical data during one hospital stay and stable COPD management data through regular clinical follow-ups; the observing parameters were identified and selected by all the principle investigators from the collaborating centers together with a panel of national experts. A paper case report form was designed to collect research information bedside, and an electronic case report form was designed to upload the research information to the study Electronic Data Capture (EDC) system.

For each patient, a baseline survey was conducted within 1–3 days after hospitalization to collect information on medical history, physical examination, and inpatient diagnosis. During the hospital stay, information on questionnaires [CAT questionnaire, PEACE questionnaire (consisting of eight questions assessing daily variance of COPD symptoms, i.e., dyspnea, purulent sputum, sputum volume, upper respiratory tract infection, fever, wheeze, cough, breath rate), mMRC questionnaire], medical examinations, laboratory tests, and treatments was recorded. Comorbidities including respiratory diseases (pneumonia, pulmonary embolism, interstitial lung disease, pulmonary arterial hypertension, lung cancer, asthma, respiratory failure), cardiovascular diseases (myocardial infarction, angina pectoris, hypertension, chronic heart failure, ventricular premature beat, right bundle branch block, atrial fibrillation), metabolic diseases (diabetes, osteoporosis), and digestive diseases (gastroesophageal reflux disease, peptic ulcer, cirrhosis), as well as malignancies other than lung cancer, peripheral arterial disease, venous thromboembolism, cerebrovascular disease, anxiety/depression, musculoskeletal dysfunction, chronic kidney disease, etc. were recorded. When a patient was discharged from hospital, information on diagnosis, CAT questionnaire, disease prognosis, intensive care unit stay (if any), and costs was collected. During each follow-up visit, information on the management; progress; recurrence; and prognosis of COPD, pharmacological, and non-pharmacological treatment was collected; meanwhile, scale and questionnaire surveys [CAT, mMRC, SGRQ, HADS] were conducted. An overview of the collected data is listed in Supplementary Table 1.

The primary aim of the study was to estimate the mortality rate during hospital stay; the sample size required is 6080 individuals, which was calculated based on an estimated mortality rate of 5% with an absolute precision of 2%, 95% confidence interval, and high design effect when recruiting patients from 40 hospitals, using the software CSurvey 2.0^12^; and finally 7600 individuals are required with an assumption of 20% drop-outs, as loss to follow-up is inevitable in such longitudinal design.

## Supplementary information

Supplementary Table 1

## Data Availability

No datasets were generated or analyzed during the current study.

## Code availability

No codes were generated during the current study.
