# Supplementary material for: Estimating mortality among inpatients with acute exacerbation of chronic obstructive pulmonary disease using registry data
Source: NPJ Prim Care Respir Med. 2020 Jun 16;30:28. doi: 10.1038/s41533-020-0186-y (PMC7297959; doi:10.1038/s41533-020-0186-y)
Supplement: Supplementary file 1 — Supplementary Table 1 [file 41533_2020_186_MOESM1_ESM.pdf]

Supplementary Table 1 Observing variables of the ACURE study

Data module	Variable
Baseline Survey	
Medical history	Demographic data
	Symptoms of the current AECOPD before hospitalization
	Potential stimulus of the current AECOPD
	Treatment against exacerbation before hospitalization
	Age of first diagnosis of COPD
	Frequency of hospitalization
	Respiratory symptoms
	Disease history other than COPD
	History of surgery
	COPD management at stable stage
	Preventive medicine use
	Allergic history and occupational exposure history
	Tobacco exposure
Physical examination	Weight and height
	Heart and breath frequency
	Blood pressure and body temperature
Inpatient diagnosis	AECOPD confirmation
	Comorbidities: respiratory, cardiovascular, metabolic, digestive, cancer, anxiety/depression, etc.
Questionnaires	PEACE
	CAT and mMRC
Medical examinations	Pulmonary function test: FEV1, FVC, IC, VC, FRC, RV, TLCO, DLCO
	Blood gas analysis (record oxygen therapy and ventilation): pH, PaO2, HCO_3, Lac
	CT: diagnosis, photographic images stored with original size
	Echocardiography: diagnosis with LVEF, PASP
	V/Q SPECT: diagnosis
	Lower extremity venous ultrasound: : diagnosis
	ECG
	Pathogen examination
	Serology and urine antigen examination
	Lab test: WBC, NE%. LY%, EO%, RBC, HCT, PLT, NE, LY, EO, HGB; ALB, CHOL, AST, TBIL, ALP, BUN, Ca, Na, Glu, LDH, TG, ALT, DBIL, γ-GT, Cr, Cl, K, CK, PCT, hs-CRP, ESR, Fbg, NTproBNP, D-dimer, HbA1C, Total-IgE, hs-cTnT, hs-cTnI
Treatment	Medicine
	Route of administration
	Dose
	Starting/end date

Endpoint measurement	AECOPD controlled, discharge upon request,
	Cause of death
Diagnosis at discharge	AECOPD confirmation
	Comorbidities: respiratory, cardiovascular, metabolic, digestive, cancer, anxiety/depression, etc.
RICU/ICU stay	Ventilation types, duration
	Vasoactive drug use
Costs	Health insurance type
	Direct healthcare cost
Follow-up survey	
Endpoint measurement	Death
	Cause of death
AECOPD recurrence	Recurrence: severity assessment
	Newly diagnosed comorbidities
Re-admission	Re-admission date and duration
Treatment	Medicine
	Route of administration
	Dose
	Starting/end date
	Non-pharmacological treatment
Medical examinations	Pulmonary function test: FEV1, FVC, IC, VC, FRC,RV,TLCO, DLCO
	CT: diagnosis, photographic images stored with original size
AECOPD assessment	Dyspnea
	Sputum
	Upper respiratory tract infection
	Fever
	Wheezing
	Cough
	Breath rate or pulse rate
Scales and questionnaires	CAT and mMRC
	SGRQ
	HADS
